# JAK2/STAT3 pathway mediates neuroprotective and pro-angiogenic treatment effects of adult human neural stem cells in middle cerebral artery occlusion stroke animal models

**DOI:** 10.18632/aging.204410

**Published:** 2022-11-29

**Authors:** Geun-Hyoung Ha, Eun Ji Kim, Jee Soo Park, Ji Eun Kim, Hyun Nam, Je Young Yeon, Sun-Ho Lee, Kyunghoon Lee, Chung Kwon Kim, Kyeung Min Joo

**Affiliations:** 1 Medical Innovation Technology Inc. (MEDINNO Inc.), Seoul 08513, South Korea; 2 Department of Anatomy and Cell Biology, Sungkyunkwan University School of Medicine, Suwon 16419, South Korea; 3 Stem Cell and Regenerative Medicine Center, Research Institute for Future Medicine, Samsung Medical Center, Seoul 06351, South Korea; 4 Department of Neurosurgery, Samsung Medical Center, Sungkyunkwan University School of Medicine, Seoul 06351, South Korea; 5 Department of Health Sciences and Technology, SAIHST, Sungkyunkwan University, Seoul 06351, South Korea; 6 Single Cell Network Research Center, Sungkyunkwan University School of Medicine, Suwon 16149, South Korea; 7 Biomedical Institute for Convergence at SKKU (BICS), Sungkyunkwan University, Suwon 16419, South Korea

**Keywords:** adult human neural stem cells, middle cerebral artery occlusion, stroke, neuroprotection, pro-angiogenic effects

## Abstract

Mismatches between pre-clinical and clinical results of stem cell therapeutics for ischemic stroke limit their clinical applicability. To overcome these discrepancies, precise planning of pre-clinical experiments that can be translated to clinical trials and the scientific elucidation of treatment mechanisms is important. In this study, adult human neural stem cells (ahNSCs) derived from temporal lobe surgical samples were used (to avoid ethical and safety issues), and their therapeutic effects on ischemic stroke were examined using middle cerebral artery occlusion animal models. 5 × 10^5^ ahNSCs was directly injected into the lateral ventricle of contralateral brain hemispheres of immune suppressed rat stroke models at the subacute phase of stroke. Compared with the mock-treated group, ahNSCs reduced brain tissue atrophy and neurological sensorimotor and memory functional loss. Tissue analysis demonstrated that the significant therapeutic effects were mediated by the neuroprotective and pro-angiogenic activities of ahNSCs, which preserved neurons in ischemic brain areas and decreased reactive astrogliosis and microglial activation. The neuroprotective and pro-angiogenic effects of ahNSCs were validated in *in vitro* stroke models and were induced by paracrine factors excreted by ahNSCs. When the JAK2/STAT3 signaling pathway was inhibited by a specific inhibitor, AG490, the paracrine neuroprotective and pro-angiogenic effects of ahNSCs were reversed. This pre-clinical study that closely simulated clinical settings and provided treatment mechanisms of ahNSCs for ischemic stroke may aid the development of protocols for subsequent clinical trials of ahNSCs and the realization of clinically available stem cell therapeutics for ischemic stroke.

## INTRODUCTION

Stroke, wherein the interruption of blood flow to the brain leads to infarction of the nervous tissue, is the second and third leading cause of death and disability worldwide, respectively [1]. Ischemic stroke is the most common type of stroke, occurring in more than 80% of stroke patients [2, 3]. Blockage of blood flow in ischemic stroke causes a scarcity of nutrients and oxygen, which in turn reduces energy generation in the affected brain tissue [2]. The ischemic microenvironment then induces the uncontrolled production of reactive oxygen species, breakdown of the blood-brain barrier, and infiltration of various immune cells. Consequently, chronic excitotoxicity and neuroinflammation exacerbate brain tissue damage.

Although the pathophysiological mechanisms of ischemic stroke have been elucidated, stroke patients still depend on thrombolytic treatment or mechanical thrombectomy [4]. However, therapies should be applied within six hours of disease onset. Since most ischemic stroke patients cannot have access to appropriate intervention within the time limit, many patients have diverse neurological sequelae including motor, sensory, and cognitive deficits that persist in their lifetime. Therefore, there is an urgent unmet need for effective therapies that can address the disease mechanisms of ischemic stroke and ultimately provoke regeneration of damaged neural tissue and reversal of loss of neurological functions.

Stem cells that have differentiation capacities into functional neural cells as well as paracrine neuroprotective and anti-inflammatory effects could overcome the limitations of current therapies for ischemic stroke [5]. Several studies have reported that stem cell transplantation shows promising therapeutic potential for ischemic stroke [6, 7]. Although stem cells derived from various sources such as embryonic, fetal, and adult tissues may be utilized, several challenges to realizing stem cell therapies for ischemic stroke in clinical settings remain, including ethical concerns, side effects such as potential tumorigenicity, immune rejection after transplantation, misguided incomplete differentiation, and unclear treatment mechanisms. These challenges can also restrict the therapeutic effects of stem cell agents in ischemic stroke.

Neural stem cells (NSCs) that can be cultivated from adult temporal lobe surgical samples reportedly exhibit neuroprotective, pro-angiogenic, and neural cell-producing activities, and show significant pre-clinical therapeutic effects in animal models of spinal cord injury and ischemic stroke [8, 9]. Moreover, adult human NSCs (ahNSCs) have little tumorigenic potential and have no ethical concerns compared to other stem cell types [10]. Regarding that those characteristics of ahNSCs are appropriate to treat ischemic stroke, in this study, it was examined that ahNSCs could be utilized to treat ischemic stroke using treatment protocols that could be translated into clinical trials and middle cerebral artery occlusion (MCAO) animal models. Moreover, the treatment mechanisms of ahNSCs and the signaling pathways involved were elucidated in this study.

## MATERIALS AND METHODS

### Animals and MCAO models

Animal experimental procedures were approved by the Institutional Animal Care and Use Committee (IACUC) of the Laboratory Animal Research Center at the Sungkyunkwan University School of Medicine (SKKUIACUC2020-06-22-1). Stroke was induced by the standard middle cerebral artery occlusion (MCAO) methods [11]. For the primary culture of cortical neurons, female pregnant Sprague-Dawley (SD) rats were purchased from Orient Bio Inc. (Seongnam, South Korea). For MCAO animal models, male SD rats (250-300 g, 8 weeks old) were purchased from Daehan bio-link (Eumseong, South Korea). Animals were housed under normal conditions with 24° C, 50% relative humidity, and 12 hours (h) light/dark cycle. For MCAO surgery, rats were anesthetized with 3-5% isoflurane (Hana Pharm, Co., LTD. Hwaseong, South Korea). After the left common carotid artery (CCA) and external carotid artery (ECA) were exposed, the left ECA was permanently tied. Then, a 4-0 medium MCAO suture L910 PK10 (4037910PK10, Doccol Corporation, Sharon, MA, USA) was inserted into a hole made at the left ECA, proximal to the tie. The diameter and length of the coated suture were 0.39 and 10 mm, respectively. The suture was propagated 20-25 mm from the left ECA to the left MCA via the left CCA, to occlude the left MCA. One hour after MCAO, the vessel was re-perfused by withdrawing the suture and tying the ECA proximal to the hole. After surgery, the rats were injected with ketoprofen (Uni Biotech, Anyang, South Korea) at a dose of 5 mg/kg daily for 2 days (d). Rats in the normal control group (Control group) were anesthetized at the time of MCAO surgery.

### ahNSC primary culture and preparation of conditioned media (CM)

To acquire surgical samples for AhNSCs culture, informed written consent was obtained from each patient according to guidelines approved by the Institutional Review Board (IRB) of Samsung Medical Center (SMC, Seoul, Korea; IRB file number: SMC 2015-03-061). AhNSCs (NS18-008TL) were primarily cultured using the temporal lobe of the brain removed from a donor with focal cortical dysplasia type IIIa. The brain tissue was mechanically minced and placed in an enzyme mixture solution containing papain (10 unit/mL, P4762, Sigma-Aldrich, St. Louis, MO, USA), DNase I (0.1 mg/mL, 11284932001, Roche, Basel, Switzerland), and D, L-cysteine (4 mg/mL, C4022, Sigma-Aldrich) in Dulbecco’s modified Eagle Medium/Nutrient Mixture F-12 (DMEM/F12) medium (11330-032, Gibco, Waltham, MA, USA) for 15-30 minutes (min) at 37° C. After mild trituration, the cells were filtered through a cell strainer (40 μm, 10737821; BD Biosciences, Franklin Lakes, NJ, USA). Following Percoll (1.13 g/mL ± 0.005 g/mL, P4937, Sigma-Aldrich) purification, adherent culture on poly-L-ornithine (10 μg/mL, A-004-M, Sigma-Aldrich)-coated dishes was cultured in Dulbecco’s modified Eagle Medium/Nutrient Mixture F-12 (DMEM/F12) medium containing with 5 μg/mL gentamicin (15710-064, Gibco), 1% B-27 supplement (17504-044, Gibco), 1% N-2 supplement (17502-048, Gibco), 50 ng/mL epidermal growth factor (EGF, 236-EG-01M, R&D Systems, Minneapolis, MN, USA), 50 ng/mL basic fibroblast growth factor (bFGF, 4114-TC-01M, R&D Systems), and 0.5% Fetal Bovine Serum (FBS, 26140-079, Gibco) in a 37° C, 5% CO_2_ incubator. AhNSCs were propagated after detachment using Accutase (AT104; Innovative Cell Technologies, San Diego, CA, USA). AhNSCs at *in vitro* passage 7 (P7) were used for subsequent experiments. To prepare CM, 1 × 10^6^ ahNSCs at P7 in 30 mL culture media were seeded in a 175 cm^2^ flask. The next day, the culture medium was replaced with 30 mL DMEM/F12. After 24 h, the CM of ahNSCs was harvested and then filtered through a 0.45 μm filter (PN 4614, PALL Life Sciences, Port Washington, NY, USA).

### Cell transplantation

The cell transplantation method was previously described [12, 13]. Seven days after MCAO surgery, the rats were randomly divided into control and ahNSC groups. Animals were anesthetized with 3-5% isoflurane. After the heads were fixed on a stereotaxic apparatus (DJ-308, Daejong Lab, Seoul, South Korea), a hole was made at anterior-posterior = - 0.8 mm, medial-lateral = -1.4 mm from the bregma using a dental drill (8050, DREMEL, Racine, WI, USA). A Hamilton syringe (1702, HAMILTON, Reno, NV, USA) with a 26-gauge needle was fixed in a syringe pump (D-307651, HA Harvard, Holliston, MA, USA), and then the needle was positioned into the right lateral ventricle (LV) of the brain through the hole (dorsal-ventral = - 3.8 mm from brain surface). 5 × 10^5^ ahNSCs suspended in 20 μL of Hank’s balanced salt solution (HBSS, 14185-052, Gibco) were transplanted into the right LV with a speed of 2 μL/min for the ahNSCs group. For the control (HBSS) group, 20 μL of HBSS was injected. After transplantation, the needle was removed at a rate of 1 mm/min to prevent leakage. Ketoprofen (5 mg/kg) and cyclosporine A (5 mg/kg, EG001, CKD Pharm, Seoul, South Korea) were injected daily for 2 d and until sacrifice. The HBSS group was used as a control group to examine the treatment efficacy of ahNSCs (NSC group).

### Behavioral analysis

Behavioral tests were conducted preoperatively to measure baseline performance and repeated weekly for 4 weeks after ahNSC transplantation. Each test was conducted once a week. The rotarod test was used to evaluate motor skill learning and neuromuscular coordination [14, 15] using a rotarod device (47750, Ugo Basile Co., Milan, Italy). One week before MCAO modeling, rats were trained for consecutive 3 d to alleviate learning effects and adapt to the rotarod device. Each day, the rats ran on a 6 cm diameter rod that rotated and gradually accelerated from 5 to 40 rpm for 180 seconds (s). Each day, the falling latency time was recorded four times for each animal. The passive avoidance test was performed to evaluate the learning and memory of the rats [16, 17]. A platform with two dark rooms and a gate between the rooms (PACS-30; Scitech Korea Inc., Seoul, South Korea) was used. Each passive avoidance test was performed for 2 d. On the first day, the animals were placed in a room on the platform with the gate closed. After the animals adapted to the room for 120 s, the light was turned on for 15 s. Subsequently, the door was opened. The rats were allowed to pass to the next room to avoid brightness. When the rats moved to the dark box, the door was closed, and an electric shock (0.3 mA) was applied to the rats for 15 s. On the second day, the rats were placed on the platform using the same procedures as on the first day without electric shock. The latency time from door opening to moving to the dark room was measured. Adhesive Removal test is often utilized as a test of somatosensory function [18–20]. A small adhesive dot (9.0 mm in diameter, 20-C304A, LATECH, Seoul, South Korea) was placed in the middle of the sole of the forepaw, and the animals were placed in a cage for 1 min. Contact and removal times until they felt the sensation of adhesion and took off the sticker by using their forepaws, respectively, were measured. Each animal was tested separately for the left and right forepaws four times per test.

### 2,3,5-triphenyltetrazolium chloride (TTC) staining

28 d after transplantation, the animals were euthanized, and their brains were sliced into a series of 2 mm thick coronal sections. Same levels of coronal sections were selected from each animal and then stained by 1% TTC solution (102204234, Sigma-Aldrich) in 1× phosphate buffered solution (PBS) for 2 h at room temperature (RT). The stained sections were placed on an overhead projector film and then scanned. The percent volume of brain atrophy was measured and compared to the intact contralateral hemisphere using Image J software (National Institute of Health, MD, https://imagej.nih.gov/ij/index.html); brain atrophy volume (%) = [(non-atrophy hemisphere) - (atrophy hemisphere)] / (non-atrophy hemisphere) × 100. For each animal, the average percentage volume of brain atrophy was calculated from six slices.

### Immunofluorescence (IF)

For histological analysis, the coronal brain sections were fixed in 25 mL 4% paraformaldehyde (PFA, CNP015-1000, CELLNEST, Hanam, South Korea) at 4° C for 3 d, immersed in 40 mL 30% sucrose (101809, SHINYO PURE CHEMICALS CO., LTD, OSAKA, Japan) until they completely sunk at 4° C, embedded in optimal cutting temperature compound (4583, Tissue-Tek, Tokyo, Japan), and then made to frozen blocks. 12 μm thick frozen sections were made using a cryostat (CM1850, Leica, Wetzlar, Germany), which were attached to glass slides (200826, MUTO PURE CHEMICALS CO., LTD, Tokyo, Japan). Rat brain sections were washed with 1× PBS three times for 5 min, incubated with 50% ethanol at RT for 30 min, and then treated with 2.5% normal goat serum (S-1012, Vector Laboratories, Inc., Newark, CA, USA) at RT for 1 h. Primary antibodies diluted in 1× PBS were applied to the slides at 4° C overnight: cleaved-caspase 3 (CC3) (1:400, 9661S, Cell Signaling Technology, Danvers, MA, USA), CD31 (1:50, ab28364, Abcam, Cambridge, UK), neuronal nuclear protein (NeuN) (1:100, ab177487, Abcam), glial fibrillary acidic protein (GFAP) (1:100, V2129, NSJ Bioreagents, San Diego, CA, USA), and ionized calcium-binding adapter molecule 1 (Iba1) (1:500, ab178846, Abcam). After incubation with 1% normal goat serum for 15 min, appropriate secondary antibody was applied at RT for 2 h. Nuclei were visualized by treatment with 4’,6-diamidino-2-phenylindole (DAPI) solution (1:100,000 in 1× PBS, D9564, Sigma-Aldrich) at RT for 5 min. Three parts of the cerebral cortex of the ipsilateral infarct or contralateral normal hemisphere were randomly selected and analyzed using a Leica TCS SP8 HyVolution confocal microscope (Leica Microsystems, GmbH, Mannheim, Germany). The number of CC3-, CD31-, NeuN-, GFAP-, and Iba1-positive cells was counted using ImageJ software. The average number of three regions per animal was calculated.

### Primary culture of cortical neurons and oxygen glucose deprivation (OGD) model

Cerebral cortex was separated from 18 days old SD rat embryos at 4° C in 1× HBSS, which was treated with 0.25% trypsin-EDTA (25200-072, Gibco) at 37° C for 2 min. Resulting pellets were suspended in DMEM (10-013-CV, CORNING, Corning, NY) supplemented with 1% penicillin/streptomycin (17-602E, Lonza, Quakertown, PA) and 10% FBS and then filtered through a 70 μm cell strainer (352350, BD Falcon, Franklin Lakes, NJ, USA). Cells in pass trough were seeded on Poly-D-Lysine (PDL, 50 μg/mL, A38904-01, Gibco)-coated 96 wells plate (30096, SPL, Tampa, FL, USA) at 1.5 × 10^5^ cells/well, 24 wells plate (30024, SPL) at 2.5 × 10^5^ cells/well, or 60 mm^2^ dish (20060, SPL) at 4 × 10^6^ cells/dish. The plates were coated with the PDL for 1 d. Cells were maintained at 37° C under 5% CO_2_. Four hours after cells were seeded, half of media was changed with neurobasal medium supplemented with 1% penicillin, B-27, and L-glutamine (25030-081, Gibco). The next day, half of the media was changed to neurobasal media again, and from the third day, the media was changed every 2 days. Seven days after primary culture of cortical neurons, culture media were replaced with DMEM glucose-free neurobasal media (11966-025, Gibco), and culture plates were placed in a humidified incubator (CO48JI401247, Eppendorf AG, Hamburg, Germany) at 37° C in 5% CO_2_ and 1% O_2_ for 1 h to make OGD model. Immediately after OGD, culture medium was replaced with DMEM/F12 (incomplete media, IM), CM of ahNSCs (NSC-CM), or NSC-CM with AG490 (50mM, T3434, SIGMA-ALDRICH) and cells were maintained at 37° C in 5% CO_2_ for 24 h.

### 3-(4,5-dimethylthiazolyl-2)-2,5-diphenyltetrazolium bromide (MTT) assay

Cell viability was measured using an MTT assay (298-93-1, Sigma-Aldrich). MTT solution (10 μL of MTT solution was added to each well of a 96-well plate at 37° C for 3 h, and 100 μL of dimethyl sulfoxide (DMSO, D2650-100ML, SIGMA-ALDRICH) was added to each well at 37° C for 30 min. Absorbance was measured at 570 nm using an auto X Mark microplate reader (10319, BIO-RAD, Hercules, CA, USA). Relative cell viability (%) was calculated as the relative difference in absorbance compared to the control (without OGD): (absorbance/absorbance of control) × 100.

### Immunocytochemistry (ICC)

Cells were treated with 4% PFA for 10 min, 0.1% Triton X-100 (X-100, Sigma-Aldrich) + 1% bovine serum albumin (BSA, A2153, Sigma-Aldrich) for 10 min at RT, and then primary antibody diluted in 0.1% triton X-100 + 1% BSA at 4° C, overnight; mouse anti-MAP2 (1:500, ab11267, Abcam), rabbit anti-MAP2 (1:500, ab32454, Abcam), mouse anti-NeuN (1:500, MAB377, EMD Millipore, Burlington, MA, USA), rabbit anti-NeuN (1:500, ab177487, Abcam), Tuj1 (1:500, 801202, Biolegend, San Diego, CA, USA), GFAP (1:500, V2129, NSJ Bioreagents, San Diego, CA, USA) or p-STAT3(1:100, ab76315, Abcam). After washing three times with 1× PBS, the samples were incubated with appropriate secondary antibodies at RT for 2 h: Alexa Fluor 488- or 594-conjugated goat anti-mouse or anti-rabbit IgG (1:1000, A11001, A11005, A11008, A11012, Life Technologies, Carlsbad, CA, USA). TUNEL staining was performed using an *In situ* Cell Death Detection kit (12156792910, Roche) according to the TMR Red protocol. Briefly, 50 μL of TUNEL reaction mixture was applied to each sample at 37° C for 1 h. Nuclei were visualized by treatment with a DAPI solution at RT for 5 min. ICC products were observed in three randomly chosen parts using confocal laser microscopy. Given that no immunoreactivity was confirmed in the negative controls, signal-positive cells were counted using the ImageJ analysis software.

### Western blotting analysis

Brain tissues or primary cortical neurons were lysed in RIPA lysis buffer (sc-24948, ChemCruz, TX, USA). The amount of protein in the samples was quantified using Bradford Protein Assay Dye (5000006, BIO-RAD, Hercules, CA, USA). Equal amounts of protein were loaded in OneGel PAGE kit Plus (MP014, BIOSOLUTION, Suwon, South Korea) and then separated in 1× Tris-Glycine-SDS Buffer (CBT3141, DYNE BIO, Seongnam, South Korea). The separated proteins were transferred onto a nitrocellulose membrane in 1× Tris-glycine buffer (CBT3291, DYNE BIO) with methanol (5558-4410, DAEJUNG, Siheung, South Korea). Protein separation and transfer were performed using a Tetra Vertical Electrophoresis and Blotting System (BIO-RAD). The membranes were blocked with 5% skim milk (CNS109-0500, CellNest) in Tris-Buffered saline (CBT3051, DYNE BIO) containing Tween-20 (0777-1L, VWR LIFE SCIENCE, Radnor, PA, USA) at RT for 1 h. The membranes were incubated with primary antibodies in 5% BSA at 4° C overnight: Bax (1:1,000, sc-7480, Santa Cruz Biotechnology, Dallas, TX, USA), Bcl-2 (1:100, sc-7382, Santa Cruz Biotechnology), β-actin (1:1,000, sc-47778, Santa Cruz Biotechnology), Caspase 3 (1:1,000, 9662, Cell Signaling), Cleaved-caspase 3 (1:1,000, 9664, Cell Signaling), p-JAK2(1:500, 3776S, Cell Signaling), JAK2 (1:500, 3230S, Cell Signaling), p-STAT3 (1:1,000, 9145S, Cell Signaling), and STAT3 (1:1,000, 30835S, Cell Signaling). The membranes were incubated with horseradish peroxidase (HRP)-conjugated goat anti-mouse (SA001-500, GenDEPOT, Barker, TX, USA) or rabbit IgG secondary antibodies (1:10,000, SA002-500, GenDEPOT) in Tris-buffered saline with Tween-20 at RT for 1 h. A detection kit solution (LF-QC0101, GWVITEK, Seoul, South Korea) was applied to the membranes, and the signals were detected using X-ray films (AGFA, Mortsel, Belgium) or a detection system (JP-33, JPI Healthcare Co., Ltd., Seoul, South Korea). Bands were quantified using the ImageJ software and normalized to β-actin.

### Microarray and quantitative real-time polymerase chain reaction (qRT-PCR)

Primary cortical neurons were collected using scrappers and centrifuged at 13,000 rpm for 1 min. The pellet was analyzed by microarray, according to the procedure described by Macrogen Co. (Seoul, South Korea). Briefly, total RNA identical to that used in the SAGE protocol was used to generate cDNA. cDNA was synthesized using the GeneChip WT (Whole Transcript) Amplification Kit (902280, Thermo-Fisher Scientific, Uppsala, Sweden), as described by the manufacturer. The sense cDNA was then fragmented and biotin-labeled with terminal deoxynucleotidyl transferase using the GeneChip WT Terminal labeling kit (900670, Thermo-Fisher Scientific). Approximately 5.5 μg of labeled DNA target was hybridized to the GeneChip™ Rat Gene 2.0 ST Array (902124, Thermo-Fisher Scientific) at 45° C for 16 h. Hybridized arrays were washed and stained on a GeneChip Fluidics Station 450 and scanned on a GCS3000 Scanner (00-0210, Thermo-Fisher Scientific). Probe cell intensity data computation and CEL file generation were performed using Affymetrix^®^ GeneChip Command Console^®^ Software (AGCC). Data were summarized and normalized using the robust multi-average (RMA) method implemented in the Affymetrix^®^ Power Tools (APT). Results were exported with gene-level RMA analysis, and differentially expressed gene (DEG) analysis was performed. Genes with a fold change ≥ 2 between groups were identified as DEGs. For each DEG set, hierarchical cluster analysis was performed using complete linkage and Euclidean distance as measures of similarity. Gene enrichment and functional annotation analysis for the significant probe list was performed using Gene Ontology (GO, http://geneontology.org). GO enrichment analysis was performed using the Database for Annotation, Visualization, and Integrated Discovery (DAVID). All data analyses and visualization of DEGs were conducted using R 3.3.2 (https://www.r-project.org).

qRT-PCR was performed to confirm the microarray results. Total RNAs was extracted from the cells using a RNeasy RNA extraction Mini kit (74004, Qiagen Sciences, Germantown, MD, USA). Reverse transcription was performed using an EasyScript™ cDNA Synthesis Kit (G236, Applied Biological Materials Inc., Richmond, Canada) using Oligo (dT) primers. For qRT-PCR analysis, a Rotor-Gene Q real-time PCR detection system (9001861, Qiagen Sciences) and FastStart Essential DNA Green Matser (06402712001, Roche) were used as follows:95° C for 5 min and 30 s, and 40 cycles (15 s at 95° C, 1 min at 60° C). The data were analyzed with a normalized gene expression method using the Rotor-Gene Q software (Qiagen Sciences), and *β*-actin was used as a reference for normalization. The gene-specific primer sequences are listed in Supplementary Table 1.

### *In vitro* tube formation assay

We added 100 μL of Matrigel solution (BD Biosciences, 356231) to the wells of 96-well plates and incubated at 37° C for 30 min. 2.0 × 10^3^ Human umbilical vein endothelial cells (HUVECs, C-12200, PromoCell, Heidelberg, Germany) in 200 μL of endothelial cell basal medium (C-22211, PromoCell) supplemented with growth medium supplement mix (C-39216, PromoCell) and penicillin-streptomycin were seeded on the Matrigel in 96-well plates, and then incubated at 37° C in 5% CO_2_ and 1% O_2_ for 1 h to have OGD challenge. After OGD, the medium was changed to IM, NSC-CM, or NSC-CM with AG490 (50 μM). After 24 h, the total tube length was measured using the ImageJ software.

### Transwell migration assay

HUVECs (1 × 10^4^ cells/well) in endothelial cell basal medium supplemented with growth medium supplement mix and penicillin-streptomycin were seeded on 24-well clear flat-bottom TC-treated Multiwell Cell Culture Plates (353047, Falcon, Glendale, AZ). After 1 h of OGD, the medium was replaced with IM, NSC-CM, or NSC-CM with AG490 (50 μM). After incubation at 37° C for 24 h, cells that did not migrate through the membrane were removed. The cells migrated were fixed with 4% PFA for 10 min and then stained with 0.2% crystal violet for 30 min. The stained cells were counted in three randomly selected areas using ImageJ software.

### Statistical analysis

All data are presented as mean ± standard error of the mean (SEM) or standard deviation (SD). Statistical analyses were performed using Prism 9 software (GraphPad Software, Inc., San Diego, CA, USA). Two-tailed Student’s t-test assuming equal variance, one-way analysis of variance (ANOVA) + Tukey’s post hoc analysis were used for comparisons between two samples and among multiple groups, respectively. Statistically significant differences are indicated by asterisks: * *P* < 0.05, ** *P* < 0.01, *** *P* < 0.001.

## RESULTS

### *In vitro* characteristics of ahNSCs

AhNSCs were primarily cultured from adult temporal lobe surgical samples under adherent culture conditions, as previously described, and demonstrated the expected bipolar morphologies [21–23]. We confirmed the expression of surface markers of ahNSCs and *in vitro* differentiation potential into multiple types of neural cells using flow cytometry and ICC, respectively. Primarily cultured ahNSCs expressed NSC markers such as nestin, CD29, CD44, and CD140b, while hematopoietic stem cell markers, including CD11b, CD19, CD31, and CD45, and the negative control (HLA-DR) were not detected (Supplementary Figure 1A). Nestin expression was also observed in almost every ahNSC before differentiation by ICC, which was significantly reduced under differentiation conditions (Supplementary Figure 1B). The differentiated ahNSCs showed various morphologies associated with differentiated neural cells and expressed lineage-specific markers of differentiated neural cells, such as Tuj1 (neurons), GFAP (astrocytes), and Claudin11 (oligodendrocytes) (Supplementary Figure 1B). These results indicate that ahMNCs retain the essential characteristics of NSCs, including self-renewal potential, expression of NSC-specific markers, and capacity for differentiation into multiple neural cell lineages.

### Significant therapeutic effects of ahNSCs for ischemic stroke

**Figure 1:**
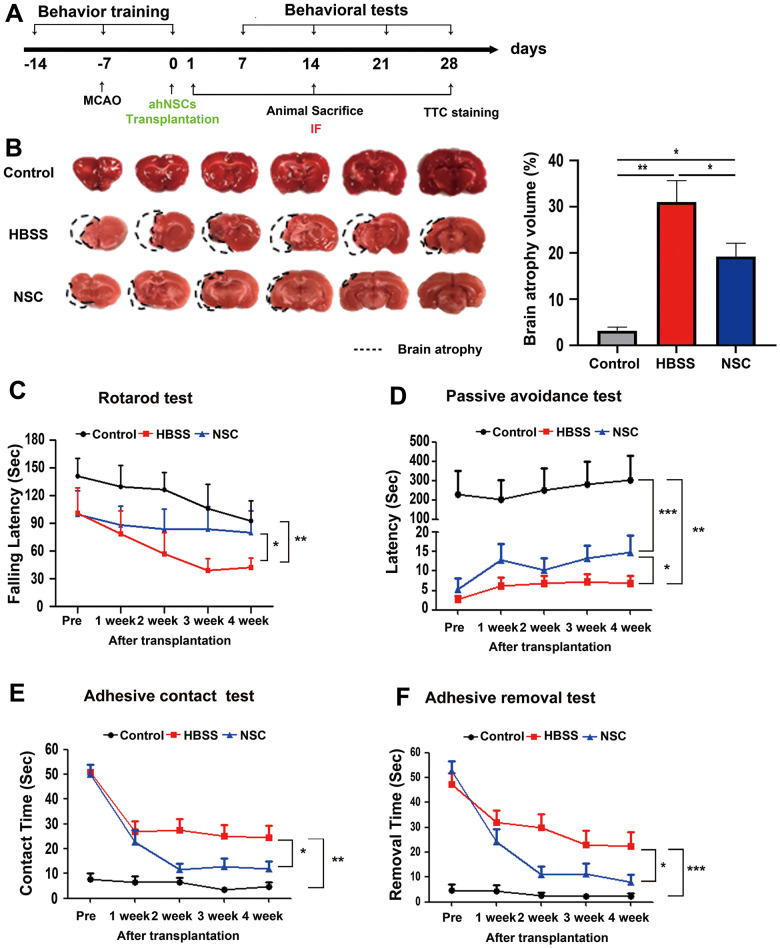
**Significant therapeutic effects of ahNSCs for ischemic stroke.** (**A**) *In vivo* experimental procedure was illustrated. (**B**) Left. Representative images of TTC staining at 28 d after ahNSCs transplantation (n = 3 for the control group, n = 7 for the HBSS group, n = 9 for the NSC group). The black dashed lines denote the atrophic regions of brains. Right. Brain atrophy volumes (%) were calculated and compared. Mean ± SEM. * *P* < 0.05; ** *P* < 0.01. (**C**) Rotarod test measured time to fall from rotating rotarod which accelerated from 5 to 40 rpm for 180 s. Each day, four latency times were averaged for each animal and compared (n = 7 for the control group, n = 6 for the HBSS group, n = 6 for the NSC group). Mean ± SEM. * *P* < 0.05; ** *P* < 0.01 at 28 d after ahNSCs transplantation. (**D**) Memory function, latency time to avoid pain stimuli using space clues, was analyzed by passive avoidance test (n = 6 for the control group, n = 15 for the HBSS group, n = 17 for the NSC group). Mean ± SEM. * *P* < 0.05; ** *P* < 0.01; ***P <0.001 at 28 d after ahNSCs transplantation. (**E**, **F**) In adhesive-removal test, contact (**E**) and removal (**F**) time until animals feel the sensation of adhesion and take off sticker by using their forepaws, respectively, was accessed (n = 13 for the control group, n = 23 for the HBSS group, n = 24 for the NSC group). Mean ± SEM. * *P* < 0.05; ** *P* < 0.01; ***P <0.001 at 28 d after ahNSCs transplantation.

Previous studies have suggested that transplantation of NSCs in the early phase of stroke can improve the behavioral functions of animals with ischemic stroke [24, 25]. Accordingly, in the present study, the time point of transplantation of ahNSCs was chosen as 7 d after the induction of ischemic stroke in the left brain hemisphere of rats, which corresponds to the subacute phase [26]. AhNSCs (5 × 10^5^ cells) in 20 μL HBSS were injected into the contralateral right LV. The experimental procedure used in this study is shown in Figure 1A. The transplanted ahNSCs were detected in the stroke lesions by both immunohistochemistry against human cytoplasm (Supplementary Figure 2A) and PCR of the human specific-Alu sequence (Supplementary Figure 2B) at 1 day after injection, which indicated the lesion-tropism and survival of ahNSCs. The therapeutic effects of ahNSCs were determined by changes in infarct volume (TTC staining) at 28 d after ahNSC transplantation and sensorimotor (rotarod and adhesive-removal test) and memory (passive avoidance test) function tests from 7 to 28 d after ahNSC transplantation. Infarction volume decreased significantly in the ahNSC-transplanted (NSC) group (n = 9) compared with the Sharm-treated (HBSS) group (n = 7) (HBSS 31.05 ± 12.21 vs. NSC 19.23 ± 8.66 %) (Figure 1B). In the rotarod test, all animals with ischemic stroke (HBSS and NSC groups) showed a decrease in motor function compared to the control group (n = 7). However, a significant improvement in motor function was observed in the NSC group (n = 6) compared to that in the HBSS group (n = 6). At 28 d after ahNSC transplantation, the animals of the NSC group remained on the rotating rod significantly longer than those of the HBSS group (HBSS and NSC group: 46.20 ± 9.69 and 77.36 ± 19.99 s, respectively) (Figure 1C). In line with this, memory to avoid pain, the latency to enter the dark chamber in the passive avoidance test, of the NSC group (n = 17) was significantly longer at 28 d after ahNSCs transplantation compared to that of the HBSS group (n = 15) (HBSS and NSC group: 6.09 ± 1.81 and 14.77 ± 4.38 s, respectively) (Figure 1D). The adhesive-removal test showed that ahNSCs preserved the sensorimotor functions of animals with ischemic stroke. The HBSS and NSC groups showed increased delays in time-to-contact (Figure 1E) and time-to-remove (Figure 1F) adhesive tape at all the time points tested. Compared to the HBSS group (n = 23), the rats transplanted with ahNSCs (n = 24) performed better at 14, 21, and 28 d after ahNSC transplantation. On day 28, there was a significant difference between the NSC and HBSS group (HBSS and NSC group: 24.6 ± 4.6 vs. 12.0 ± 2.8 s for time-to contact, 22.4 ± 5.7 vs. 7.9 ± 3.2 s for time-to remove). Altogether, ahNSCs had significant therapeutic effects in ischemic stroke, reducing infarct volumes, and improving various neurological functions in MCAO animal stroke models.

### Neuroprotective and pro-angiogenic treatment mechanisms of ahNSC

**Figure 2:**
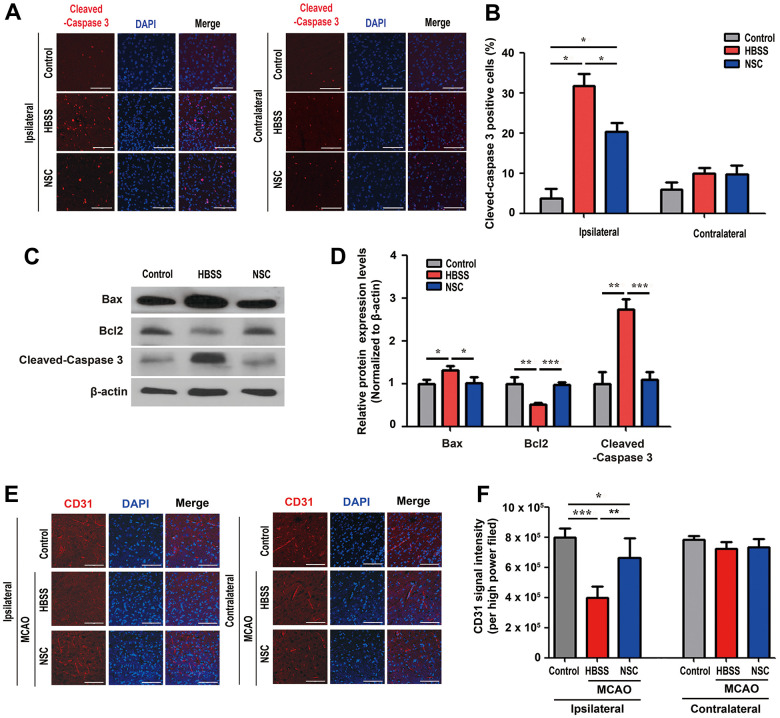
**Neuroprotective and pro-angiogenic activities of ahNSCs.** (**A**) Immunofluorescence for cleaved-caspase 3 was performed at 1 d after ahNSCs transplantation. Representative images show the immunostaining results of cleaved-caspase 3 (red) in the ipsilateral (infarct) area and contralateral (non-infarct) areas of each group (n = 3 for the control group, n = 3 for the HBSS group, n = 4 for the NSC group). Scale bar = 100 μm. (**B**) Percent of cleaved-caspase 3-positive cells were calculated and compared among the groups. Mean ± SEM. * *P* < 0.05. (**C**) Expression of Bax (23 kDa), Bcl-2 (26 kDa), and cleaved-caspase 3 (17/19 kDa) in the brains of ischemic stroke animal models (n = 3 for the control group, n = 3 for the HBSS group, n = 4 for the NSC group) was accessed by western blot analysis. The pictures show representative images. β-actin (43 kDa) = loading control. (**D**) Relative protein expression levels of Bax, Bcl-2, and cleaved-caspase 3 was calculated and compared among the groups. Mean ± SD. * *P* < 0.05; ** *P* < 0.01; *** *P* < 0.001. (**E**) Immunofluorescence for endothelial cells marker, CD31, was performed at 28 d after ahNSCs transplantation. Representative images of the ipsilateral (infarct) or contralateral (non-infarct) areas of each group (n = 5 for the control group, n = 6 for the HBSS group, n = 5 for the NSC group) are shown. Scale bar = 100 μm. (**F**) The CD31 signal intensity per high power field was measured and then compared. Mean ± SEM. * *P* < 0.05; ** *P* < 0.01; *** *P* < 0.001.

In the spinal cord injury animal models, ahNSCs exert their therapeutic effects via neuroprotective and pro-angiogenic paracrine activities [27, 28]. To elucidate the neuroprotective effects of ahNSCs in cerebral ischemic injury, we first examined immunofluorescence with an apoptosis marker, cleaved-caspase 3 (Figure 2A), and TUNEL assay (Supplementary Figure 3) 1 d after ahNSC transplantation in MCAO animal models. We observed significant reduction in the population of cleaved-caspase 3-positive cells in the NSC (20.4 ± 4.4 %) group (n = 4) compared to the HBSS (31.7 ± 5.1 %) group (n = 3) at the ipsilateral infarct area (Figure 2B). This reduction was verified by western blot analysis using anti-Bax, Bcl-2, and cleaved-caspase 3 antibodies. Western blot analysis showed that the expression levels of Bax, a pro-apoptotic factor, and cleaved-caspase 3 were significantly reduced, whereas the expression of Bcl-2, an anti-apoptotic factor, was significantly induced in the NSC group (n = 4) compared to the HBSS group (n = 3) 28 d after ahNSC transplantation (Figure 2C, 2D).

Angiogenesis is positively correlated with the recovery of neurological function after stroke [29]. Next, we examined whether ahNSCs promoted angiogenesis in a rat stroke model. The number of microvessels was analyzed in the ipsilateral (infarct) and contralateral (non-infarct) areas 28 d after ahNSC transplantation by CD31 immunostaining (Figure 2E). In the infarct area, CD31 immunoreactivity was significantly higher in the NSC group (n = 5, 661501.8 ± 133002.3) than in the HBSS group (n = 6, 397401.0 ± 74342.9) (Figure 2F). Together, these results suggest that ahNSCs protect damaged neural tissue, enhance angiogenesis in MCAO animal models, and exert significant therapeutic effects in ischemic stroke.

### Anti-gliosis and anti-inflammatory effects of ahNSCs in ischemic brain tissue

**Figure 3:**
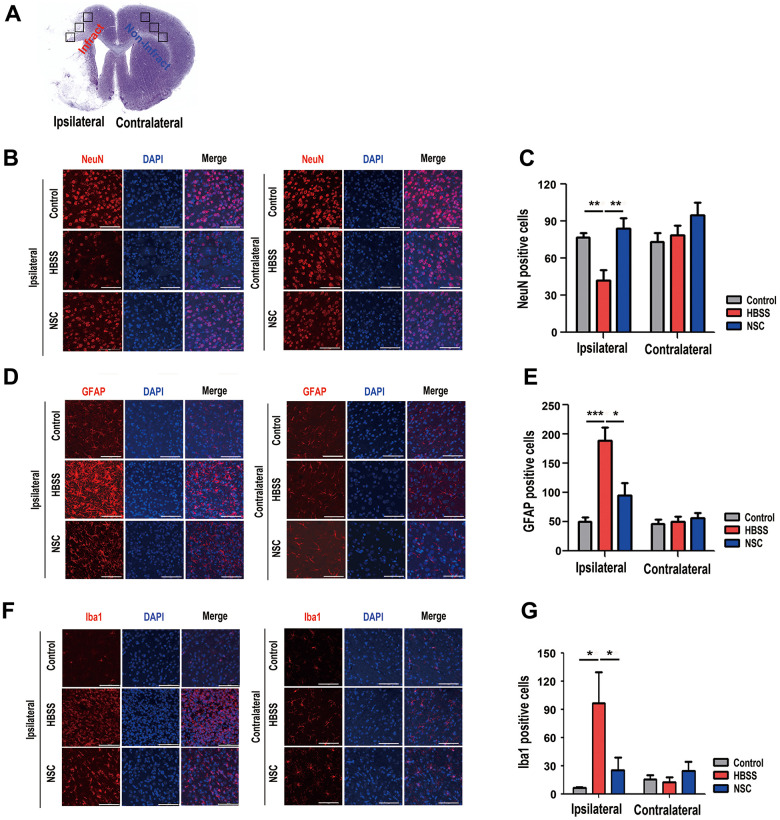
**Effects of ahNSCs on density of neurons, astrogliosis, and microglial activation.** (**A**) Ipsilateral infarct and contralateral non-infarct areas examined for each animal are illustrated. (**B**) The density of neurons was examined by immunofluorescence against NeuN at 28 d after ahNSCs transplantation. Representative images illustrate each group (n = 5 for the control group, n = 6 for the HBSS group, n = 5 for the NSC group). Scale bar = 100 μm. (**C**) The numbers of NeuN-positive cells were calculated and then compared. Mean ± SEM. ** P < 0.01. (**D**) Astrogliosis was examined by immunofluorescence against GFAP at 28 d after ahNSCs transplantation. Representative images illustrate each group (n = 5 for the control group, n = 6 for the HBSS group, n = 5 for the NSC group). Scale bar = 100 μm. (**E**) The numbers of GFAP-positive cells were calculated and then compared. Mean ± SEM. * P <0.05; *** P < 0.001. (**F**) Microglial activation was examined by immunofluorescence against Iba1 at 28 d after ahNSCs transplantation. Representative images illustrate each group (n = 5 for the control group, n = 6 for the HBSS group, n = 5 for the NSC group). Scale bar = 100 μm. (**G**) The numbers of Iba1-positive cells were calculated and then compared. Mean ± SEM. * P <0.05.

To examine the neuroprotective and pro-angiogenic effects of ahNSCs on ischemic brain tissue, brain sections of ischemic stroke animal models were immuno-stained 28 d after ahNSC transplantation. The coronal section of the normal brain shows three parts (black squares) of the ipsilateral (infarct) and contralateral (non-infarct) areas stained for each animal (Figure 3A). The number of NeuN-positive neurons decreased significantly in the HBSS group (n = 6, 44.6 ± 9.5) compared to the NSC group (n = 5, 84.4 ± 8.1) in the ipsilateral brain hemisphere, which confirmed the neuroprotective effects of ahNSCs. There was no difference in the number of neurons in the contralateral hemisphere between the three groups (Figure 3B, 3C). As previously reported [28, 30], increase in the number of GFAP (a marker of reactive gliosis) (Figure 3D, 3E)- and Iba1 (a marker of activated microglia) (Figure 3F, 3G)-positive cells was observed in the HBSS groups (n = 6, 189 ± 22.32 for GFAP and 96.53 ± 32.984 for Iba1) compared to the control group (n = 5, 50.07 ± 7.023 for GFAP and 6.55 ± 0.479 for Iba1) in ipsilateral brain hemisphere. However, they decreased significantly in the NSC group (n = 5, 94.67 ± 21.644 for GFAP and 25.467 ± 13.151 for Iba1). There was no difference in the number of GFAP- or Iba1-positive cells in the contralateral brain hemisphere among the three groups (Figure 3D–3G). Taken together, these results indicate that the neuroprotective and pro-angiogenic activities of ahNSCs further increase neuronal density and reduce astrogliosis and microglial activation in ischemic brain tissues.

### Paracrine neuroprotective effects of ahNSCs

**Figure 4:**
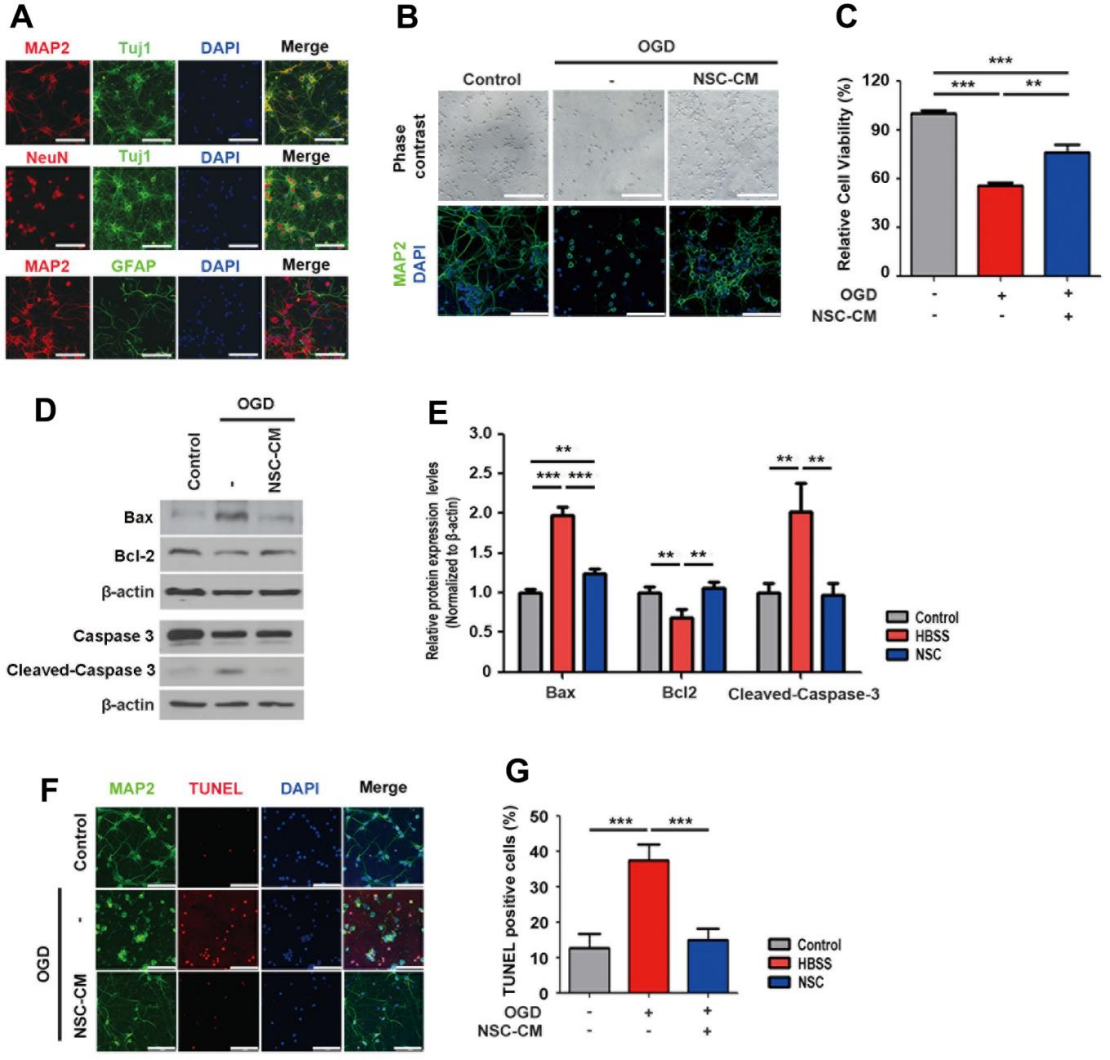
***In vitro* paracrine neuroprotective effects of ahNSCs.** (**A**) Expression of neuron (MAP2, Tuj1, NeuN)- or astrocyte (GFAP)-specific markers of primary cortical neurons were tested by ICC. Scale bar = 100 μm. (**B**) Effects of OGD condition and treatment of CM of ahNSCs (NSC-CM) on the morphology and survival of primary cortical neurons was examined by light microscope and ICC against MAP2. Scale bar = 100 μm. (**C**) Viability of primary cortical neurons was measured after OGD condition ± NSC-CM by MTT assay (n = 3 per group). Mean ± SD. ** *P* < 0.01; *** P < 0.001. (**D**) Expression of Bax (23 kDa), Bcl-2 (26 kDa), caspase 3, and cleaved-caspase 3 (17/19 kDa) of primary cortical neurons (n = 3 per group) was accessed by western blot analysis. The pictures show representative images. β-actin (43 kDa) = loading control. (**E**) Relative protein expression levels of Bax, Bcl-2, and cleaved-caspase 3 was calculated and compared among the groups. Mean ± SD. ** *P* < 0.01; *** *P* < 0.001. (**F**) Effects of OGD condition and treatment of NSC-CM on the survival of primary cortical neurons was accessed by TUNEL assay. Scale bar = 100 μm. (**G**) Percent of TUNEL-positive cell of each group (n = 3 per group) was determined and compared. Mean ± SD. ***P <0.001.

Next, we examined whether the neuroprotective effects of ahNSCs were mediated by their paracrine mediators *in vitro*. Cortical neurons were primarily cultured from the brains of rat fetuses and characterized. Primary cortical neurons expressed neuron-specific markers such as MAP2, Tuj1, and NeuN, but not the astrocyte-specific marker GFAP (Figure 4A). To simulate ischemic stroke, the OGD condition was applied to cortical neurons. In the OGD condition, primary cortical neurons were damaged to lose MAP2-positive neurites (Figure 4B) and their viability decreased significantly (n = 3 per group, 55.83 ± 1.528 vs. control, 100 ± 1.994 %) (Figure 4C). However, treatment with conditioned media of ahNSCs (NSC-CM) recovered both the damage (Figure 4B) and death (n = 3, 75.809 ± 5.109 %) (Figure 4C) of primary cortical neurons, significantly. The paracrine neuroprotective effects of ahNSCs were verified by western blot analysis of Bax, Bcl-2, and cleaved-caspase 3. Western blot analysis revealed that the expression levels of Bax and cleaved-caspase 3 were significantly reduced, whereas the expression level of Bcl-2 was significantly induced 24 h after NSC-CM treatment (n = 3) in the OGD condition compared to the HBSS group (n = 3) (Figure 4D, 4E). The *in vitro* paracrine neuroprotective effects of ahNSCs were confirmed by TUNEL staining in the OGD condition using primary cortical neurons (n = 3 per group, Figure 4F, 4G). These results indicated that the neuroprotective effects of ahNSCs are mediated by paracrine mediators.

### Role of JAK2/STAT3 in neuroprotective activities of ahNSCs

**Figure 5:**
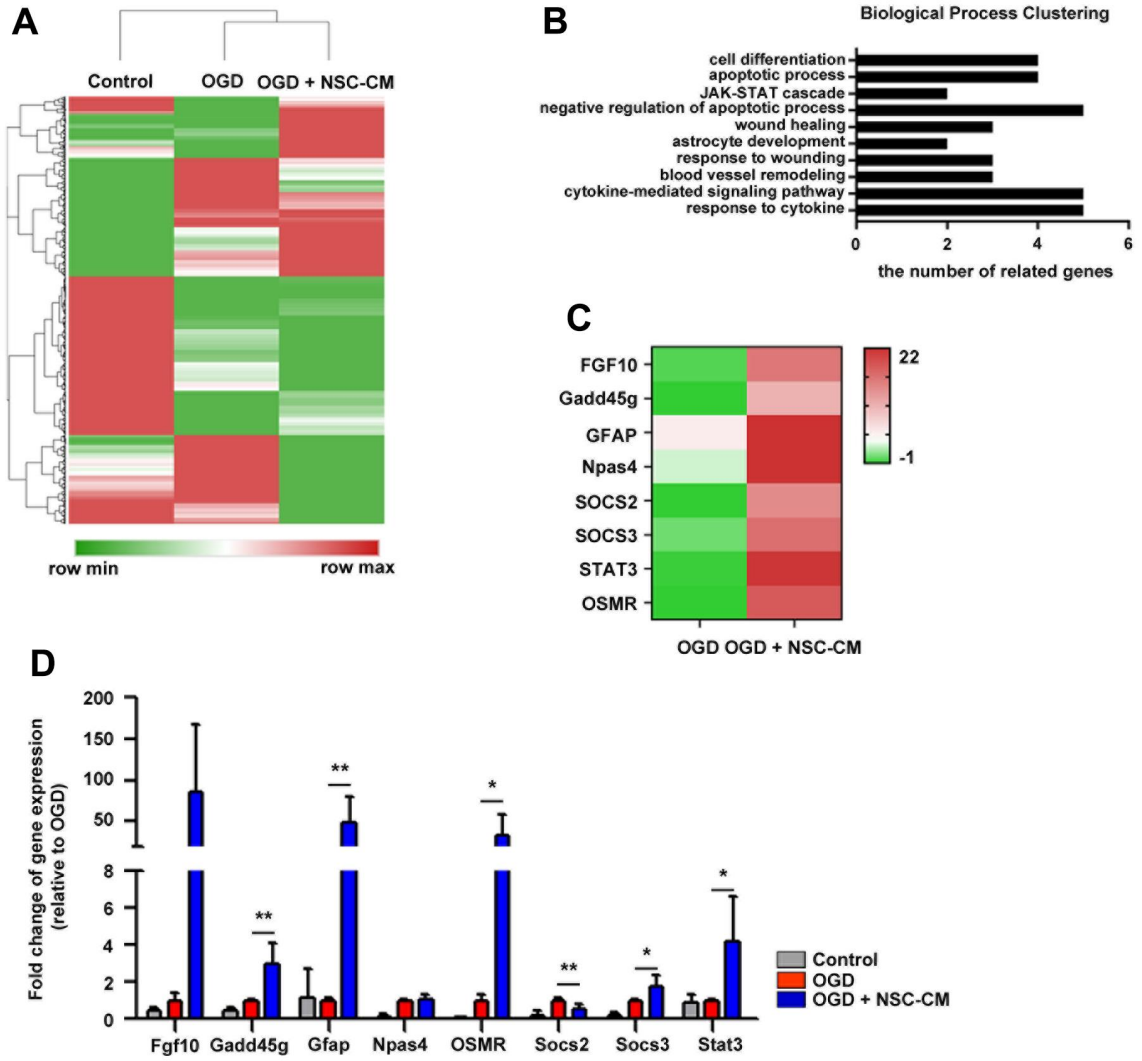
**DEGs and biological processes associated with effects of ahNSCs.** (**A**) DEGs among the primary cortical neurons in the control, OGD, and NSC-CM group was filtered out, and then hierarchical clustering was performed. (**B**) With 53 genes whose expression were up-regulated in the OGD + NSC-CM group compared to the OGD group (> 2, up-regulated genes) biological process clustering was performed. (**C**) Expression of the top eight up-regulated genes of the NSC-CM group was illustrated at heatmap. (**D**) High expression of the top eight up-regulated genes was examined by qRT-PCR (n = 3 per group). * P < 0.05 ** P < 0.01.

To investigate the mechanisms that mediate the paracrine neuroprotective activities of ahNSCs, the transcriptome of primary cortical neurons treated with NSC-CM for 24 h under OGD conditions was analyzed. The DEGs among the primary cortical neurons in the normal condition (control), in the OGD condition (OGD), and treated with NSC-CM for 24 h under OGD conditions (OGD + NSC-CM) were filtered out, and hierarchical clustering was performed (Figure 5A). Genes with a fold change value of 2 or more in the OGD + NSC-CM group compared to the OGD group were classified as up-regulated genes. The 53 up-regulated genes were further analyzed by biological process clustering (Figure 5B). The biological processes identified were negative regulation of the apoptotic process, cytokine-mediated signaling pathway, response to cytokines, cell differentiation, apoptotic process, wound healing, response to wounding, blood vessel remodeling, JAK-STAT cascade, and astrocyte development, which matched well with the treatment mechanisms of ahNSCs for ischemic stroke in this study. High expression of the top eight up-regulated genes, Fgf10, Gadd45g, GFAP, Npas4, Socs2, Socs3, Stat3, and OSMR, in the OGD + NSC-CM group (Figure 5C) was confirmed by qRT-PCR (Figure 5D). Although the GFAP expression in the stroke brain tissue was reduced by the transplantation of ahNSCs (Figure 3D), it increased significantly in the analysis of the transcriptome of primary cortical neurons. The discrepancy might originate from the different *in vitro* experimental conditions where there was no reperfusion period and few astrocytes.

**Figure 6:**
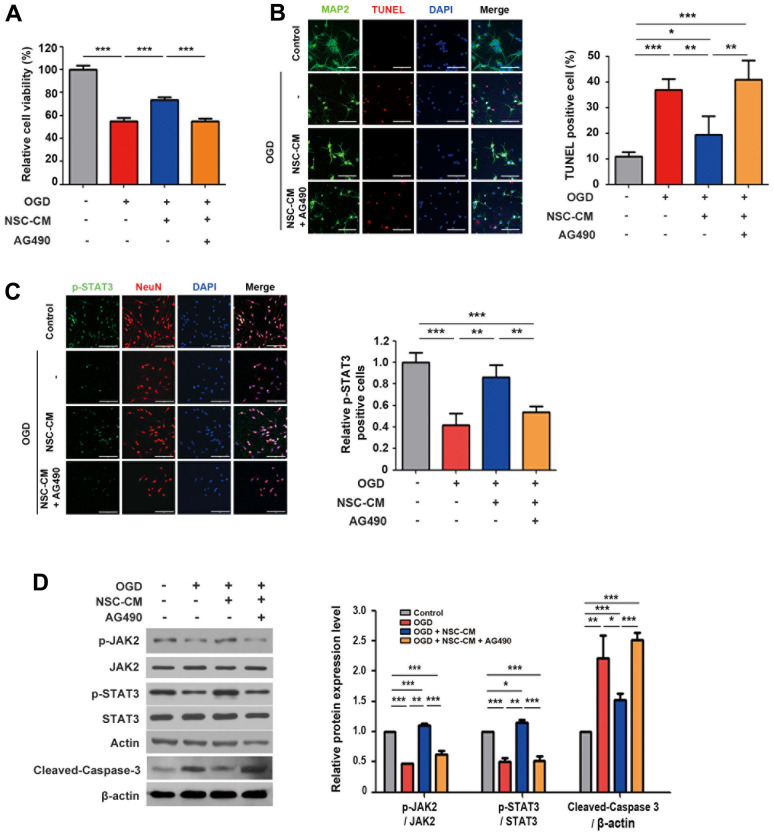
**Roles of JAK2/STAT3 signaling pathway in neuroprotective activity of ahNSCs.** (**A**) Viability of primary cortical neurons of each group was determined by MTT assay (n = 3 per group). *** *P* < 0.001. (**B**) Left. Representative images of immunofluorescence against MAP2 and TUNEL staining of primary cortical neurons (n = 3 per group). Scale bar = 100 μm. Right. Percent of TUNEL-positive cells was analyzed and compared. Mean ± SD. * *P* < 0.05, ** *P* < 0.01, *** *P* < 0.001. (**C**) Left. Representative images of immunofluorescence against p-STAT3 and NeuN of primary cortical neurons (n = 3 per group). Scale bar = 100 μm. Right. Relative ratio of p-STAT3-positive cells was analyzed and compared. Mean ± SD. ** *P* < 0.01, *** *P* < 0.001. (**D**) Left. Expression of JAK2, p-JAK2 (125 kDa), STAT3, p-STAT3 (79/86 kDa), and cleaved-caspase 3 (17/19 kDa) of primary cortical neurons (n = 3 per group) was accessed by western blot analysis. The pictures show representative images. β-actin (43 kDa) = loading control. Right. Expression of p-JAK2, p-STAT3, and cleaved-caspase 3 was normalized by JAK2, STAT3, and β-actin, respectively, and then compared. Mean ± SD. * *P* < 0.05, ** *P* < 0.01, *** *P* < 0.001.

Previous studies have suggested that JAK2/STAT3 is closely related to cerebral ischemia and can be activated during the early stages of cerebral infarction [31]. Upon JAK-STAT cascade was detected in the biological process clustering, to investigate whether the JAK2-STAT3 axis was involved in the favorable effects of ahNSCs in this study, the expression levels of STAT3 and p-STAT3 (Tyr705) in primary cortical neurons in the OGD condition were analyzed. Following OGD induction, the protein expression level of p-STAT3 (Tyr705) was significantly reduced (Supplementary Figure 4A, 4B). Moreover, we showed that the OGD group had a significantly reduced number of p-STAT3 (Tyr705)-positive cells (Supplementary Figure 4C, 4D). Next, NSC-CM-treated primary cortical neurons in the OGD condition were exposed to the JAK2/STAT3 inhibitor AG490. As shown in Figure 6A, AG490 treatment significantly and completely reversed the neuroprotective effects of NSC-CM under OGD conditions. The effect of AG490 was also observed in the TUNEL assay (Figure 6B). The NSC-CM group (n = 3, 19.5 ± 7.2 %) showed significantly reduced number of TUNEL-positive cells compared to the OGD group (n = 3, 36.9 ± 4.2 %). However, in the NSC-CM + AG490 group, the number of TUNEL-positive neurons increased (n = 3 per group, 41.0 ± 7.7 %) significantly, which was similar to that of the OGD group (Figure 6B).

As demonstrated in Figure 6C, the reduction in the viability and the increase in the number of TUNEL-positive cells in the NSC-CM + AG490 group was accompanied by decrease in the number of p-STAT3 (Tyr705)-positive primary cortical neurons (n = 3 per group, control, 100.0 ± 9.1; NSC-CM, 86.4 ± 11.5; OGD, 41.8 ± 10.6; NSC-CM + AG490, 53.6 ± 5.4 %). The roles of JAK2 and STAT3 in the neuroprotective effects of NSC-CM were further validated by western blot analysis (Figure 6D). We observed that the OGD group (n = 3) had significantly reduced expression levels of p-JAK2 and p-STAT3 (Tyr705) compared to the control (n = 3) and NSC-CM (n = 3) groups in the primary cortical neurons. Moreover, the NSC-CM + AG490 group (n = 3) showed significantly reduced levels of p-JAK2 and p-STAT3 (Tyr705) compared to those in the NSC-CM group (Figure 6D). Importantly, the NSC-CM group had significantly reduced expression of cleaved-caspase 3 compared to the OGD group, whereas the NSC-CM + AG490 group showed significantly increased level of cleaved-caspase 3 compared to the NSC-CM group (Figure 6D). Taken together, these data demonstrate that the neuroprotective effects of NSC-CM under OGD conditions may be dependent upon the activation of the JAK2/STAT3 signaling pathway.

**Figure 7:**
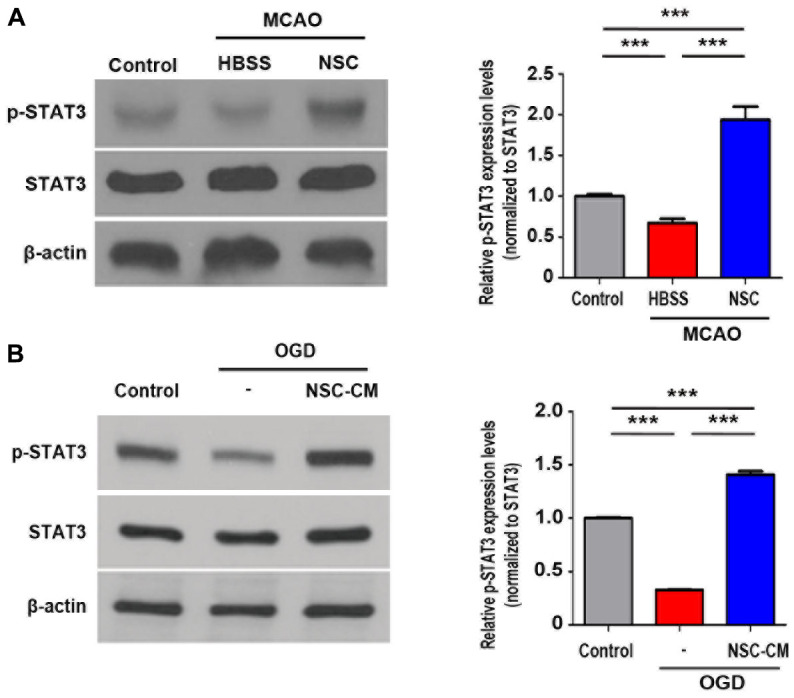
**STAT3 signaling pathway activated by ahNSCs *in vivo* and *in vitro*.** (**A**) Expression of p-STAT3 in the infarct lesion of MCAO animal models (n = 3 per group) was determined by western blotting analysis at 28 d after ahNSCs transplantation. Left. Representative images of western blot analysis are shown. β-actin (43 kDa) = loading control. Right. Expression of p-STAT3 (79/86 kDa) was normalized by STAT3 and then compared. Mean ± SD. ** *P* < 0.01, *** *P* < 0.001. (**B**) Primary cortical neurons (n = 3 per group) were cultured in OGD condition for 1 h and then incubated in IM or NSC-CM for 24 h. Expression of p-STAT3 of primary cortical neurons was determined by western blotting analysis. Left. Representative images of western blot analysis are shown. β-actin (43 kDa) = loading control. Right. Expression of p-STAT3 (79/86 kDa) was normalized by STAT3 and then compared. Mean ± SD. *** *P* < 0.001.

Finally, the phosphorylation status of STAT3 (p-STAT3, Tyr705) was tested in the brains of stroke animal models using western blot analysis. As presented in Figure 7A, transplantation of ahNSCs significantly increased the phosphorylation of STAT3 (NSC group) compared with the control and HBSS groups (n = 3 per group). Regarding the *in vitro* treatment of primary cortical neurons with NSC-CM, which significantly increased STAT3 phosphorylation (Figure 7B), the JAK2/STAT3 activation effects of ahNSCs may be mediated by paracrine mechanisms.

### Role of JAK2/STAT3 in pro-angiogenic activities of ahNSCs

**Figure 8:**
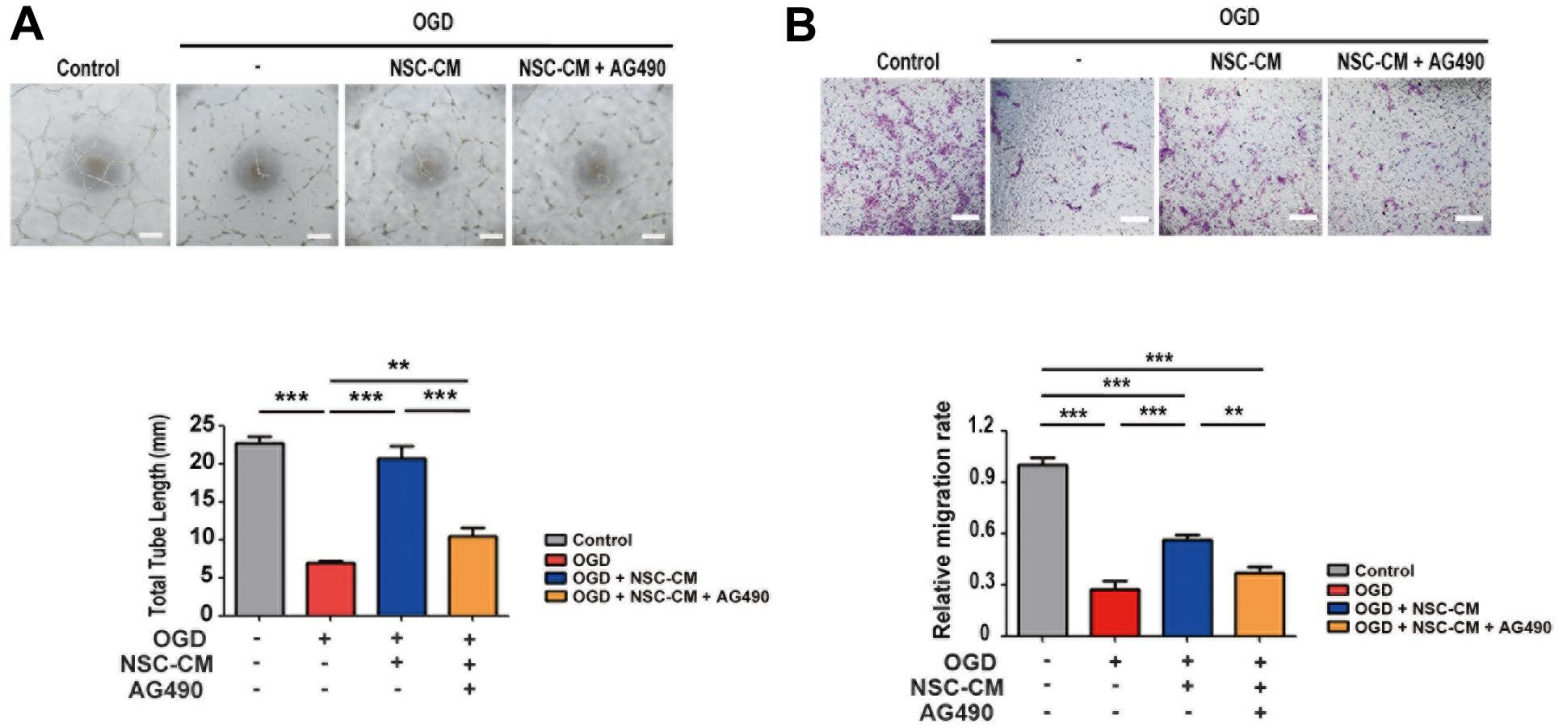
**Roles of JAK2/STAT3 signaling pathway in pro-angiogenic effects of ahNSCs.** (**A**) HUVECs were challenged by OGD for 1 h and then incubated in IM, NSC-CM, or NSC-CM with AG490 (50 μM) for 24 h. After 24 h, the total tube length was measured and compared (n = 3 per group). Scale bar = 100 μm. Mean ± SD. ** *P* < 0.01, *** *P* < 0.001. (**B**) HUVECs challenged by OGD for 1 h were cultured in IM, NSC-CM, or NSC-CM with AG490 (50 μM) for 24 h and allowed to migrate through. After 24 h, relative ratio of migrated HUVECs was measured and compared. Scale bar = 100 μm. Mean ± SD. **P <0.01; ***P <0.001.

STAT3 has been reported to be involved in angiogenesis and the migration of endothelial cells [32, 33]. Given that the pro-angiogenic activities of ahNSCs are one of the treatment mechanisms for ischemic stroke, it was determined whether the JAK2/STAT3 signaling pathway is also involved in the pro-angiogenic activities of NSC-CM using endothelial cells (HUVECs). To mimic ischemic stroke *in vitro* [34], HUVECs were subjected to OGD. In the OGD condition, HUVECs exhibited significantly decreased tube formation (Figure 8A) and migration (Figure 8B) compared to the control group in the normal oxygen and glucose environment. In the OGD condition, treatment with NSC-CM significantly improved the tube formation (Figure 8A) and migration (Figure 8B) ability of HUVECs (OGD + NSC-CM) compared to the OGD group. However, the JAK2/STAT3 inhibitor, AG490 (OGD + NSC-CM + AG490), reversed the effects of NSC-CM (Figure 8A, 8B). Collectively, these data indicated that ahNSCs may exert their pro-angiogenic effects via the JAK2/STAT3 signaling pathway.

## DISCUSSION

This study demonstrated, using *in vitro* and *in vivo* experimental models, that ahNSCs derived from adult human temporal lobe tissue exert significant therapeutic effects in ischemic stroke. The treatment effects of ahNSCs are mediated by their paracrine neuroprotective and pro-angiogenic activities, in which the JAK2/STAT3 signaling pathway may be involved. Many studies have already shown the pre-clinical therapeutic effects of various types of stem cells, including embryonic stem cells, induced pluripotent stem cells, mesenchymal stem cells, and NSCs at various differentiation statuses, for ischemic stroke [35–37]. However, no stem cell therapeutics have been clinically established for ischemic stroke [38]. One reason why clinical trials have been unsuccessful, although the same agents had significant therapeutic effects in pre-clinical experiments, might have been mismatched conditions between pre-clinical experiments and clinical trials.

Many pre-clinical studies have injected stem cells into the subcutaneous veins of animal models, from which cells are delivered to ischemic brain areas by the vascular system [39–41]. However, the collateral blood supply of the brain is more developed in rodents than in primates [42]. Therefore, the ratio of stem cells that migrate into infarction areas in animal models would be higher than that in human patients, which may affect the therapeutic effects of stem cells. Moreover, stem cells injected into the veins should pass through the capillary system of the lung before entering the systemic circulation [43, 44]. Several studies have shown that stem cells transplanted into the systemic venous system remain in the lungs instead of reaching target sites [4, 45]. In the worst case, pulmonary embolism can be induced by intravenous injection of stem cells. To better simulate the clinical setting and improve the delivery efficiency of stem cells, direct transplantation into the brain was performed in this study.

In the direct injection of stem cells into ischemic brains, the appropriate timing and location should be considered. In the acute phase of ischemic stroke (until one week after disease onset), cerebral edema could induce increased intracranial pressure (ICP), which in turn may provoke life-threatening events, such as occlusion of large intracranial arteries and brain herniation [45–47]. Therefore, it is difficult to transplant stem cells into the brain in the acute phase, although early intervention may be beneficial for therapeutic effects. Moreover, ICP is unfavorable for the survival of injected stem cells [7, 47]. There are several free spaces in the brain where stem cells can be transplanted. Among these, the cisterns and ventricles can be approached relatively safely with radiological assistance [48]. In this study, the LV of the contralateral hemisphere was chosen to maximize contact with the cerebral cortex and minimize disturbance to the infarcted area. Moreover, injections were administered in the subacute phase to avoid the acute phase, and also to transplant stem cells as soon as possible after the onset of ischemic stroke.

In this pre-clinical study, human stem cells were transplanted into a xenograft rodent model [47, 49]. However, in clinical trials, stem cell therapeutics have been allogeneic or autologous [50, 51]. This discrepancy influences the immune response of the host to therapeutics, graft survival, and therapeutic effects of stem cells. Given that secondary damage to ischemic brain tissue via inflammation and delayed apoptosis of neural cells lasts several weeks in ischemic stroke, longer survival of ahNSCs that have paracrine neuroprotective activities should be more beneficial. In previous studies using spinal cord injury animal models [52, 53], the number of ahNSCs transplanted into the damaged spinal cord decreased rapidly. More than 50% of ahNSCs were lost in the first 2 weeks after transplantation. When proper immune suppression is applied, the survival of transplanted stem cells in the brain can be improved [54]. Although cyclosporine A was used to block immune rejection after transplantation in this study, more appropriate modalities for clinical studies using allogeneic cells need to be developed to maximize the therapeutic effects of ahNSCs.

In this study, 5 × 10^5^ ahNSCs showed significant therapeutic effects in a rat model of ischemic stroke. Since 250-300 g rats were used in this study, the dose would be equivalent to 1.0-1.2 × 10^8^ cells in human stroke patients who are supposed to be 60 kg. The average brain weights of humans and rats are 1300-1400 and 2 g, respectively [55]. When the equivalent dose is calculated according to the ratio of brain weight, the human dose would be 3.25-3.5 × 10^8^ cells. There is no golden role in converting the doses of pre-clinical experiments into those of clinical trials. Therefore, the safety and efficacy of several possible dose levels should be tested in phase I and II clinical trials. Furthermore, the relationship between dose and therapeutic effects needs to be elucidated in pre-clinical settings, by which the minimum effective and saturation levels of the therapeutic agent could be derived [56, 57]. In this study, only one dose of ahNSCs was tested for the treatment of ischemic stroke. The therapeutic effects of various doses of ahNSCs should be examined further in animal models of ischemic stroke.

The neuroprotective and pro-angiogenic activities of ahNSCs were mediated by paracrine factors and involved the JAK2/STAT3 signaling pathway in *in vitro* and *in vivo* ischemic stroke models in this study. Previously, cytokines in the CM of ahNSCs have been analyzed using cytokine array analysis techniques [58]. Among the various cytokines included in the CM of ahNSCs, monocyte chemoattractant protein-1 (MCP-1, also known as CCL2) was identified as a key paracrine factor that mediates significant pre-clinical therapeutic effects of ahNSCs for spinal cord injury [27]. Since C-C chemokine receptor type 2, a receptor of MCP-1, is closely associated with JAK2, MCP-1 could also be a key paracrine mediator that induces neuroprotective and pro-angiogenic activities of ahNSCs in the ischemic stroke models in this study. Other cytokines in the CM of ahNSCs, including growth-regulated alpha protein and interleukin-6, reportedly activate the JAK2/STAT3 signaling pathway in various cell types [59–61]. Alternatively, several paracrine factors secreted by ahNSCs collaboratively exert therapeutic effects in ischemic stroke, which needs to be further elucidated.

## CONCLUSIONS

In this study, we demonstrated that ahNSCs transplanted into the brain of an ischemic stroke animal model in the subacute phase significantly reduced tissue and neurological function loss. The significant therapeutic effects of ahNSCs are mediated by their paracrine neuroprotective and pro-angiogenic activities, which may involve the activation of the JAK2/STAT3 signaling pathway. This pre-clinical study that simulates clinical settings closely and provides treatment mechanisms of ahNSCs for ischemic stroke would lead to the development of protocols for clinical trials and the realization of clinically available stem cell therapeutics for ischemic stroke.

## Supplementary Material

Supplementary Materials

Supplementary Figures

Supplementary Table 1
