# Supplementary material for: JAK2/STAT3 pathway mediates neuroprotective and pro-angiogenic treatment effects of adult human neural stem cells in middle cerebral artery occlusion stroke animal models
Source: Aging (Albany NY). 2022 Nov 29;14(22):8944–69. doi: 10.18632/aging.204410 (PMC9740376; doi:10.18632/aging.204410)
Supplement: Supplementary Materials [file aging-14-204410-s001.pdf]

SUPPLEMENTARY METHODS

Flow cytometric analysis

1 × 10^6 adult human neural stem cells (ahNSCs) in 100 µL FACS buffer (554657, BD Bioscience, Franklin Lakes, NJ, USA) were stained with monoclonal antibodies; CD31 (1:100, 558068, BD Bioscience), CD44 (1:100, 559942, BD Bioscience), CD140b (1:100, 558821, BD Bioscience), CD11b (1:100, 562793, BD Bioscience), CD19 (1:100, 555415, BD Bioscience), CD45 (1:100, 555482, BD Bioscience), or HLA-DR (1:100, 559866, BD Bioscience) at room temperature (RT) for 20 minutes (mins) in the dark. For intracellular markers, ahNSCs were treated with BD Cytofix Fixation and Permeabilization buffer (554714, BD Bioscience) at RT for 20 mins and then primary antibodies at RT for 20 mins; Nestin (1:500, 51-9007230, BD Bioscience). Cells were centrifugated at 500g for 3 mins and then resuspended in Dulbecco's phosphate-buffered saline (DPBS) with 4% paraformaldehyde (PFA). Cells were analyzed by a flow cytometer (BD Bioscience).

Immunocytochemistry (ICC)

AhNSCs were differentiated by treating IBMX (0.5mM, Gibco) in Dulbecco's modified Eagle Medium/Nutrient Mixture F-12 (DMEM/F12) medium (11330-032, Gibco, Waltham, MA, USA) containing with 5 µg/mL gentamicin (15710-064, Gibco), 1% B-27 supplement (17504-044, Gibco) and 1% N-2 supplement (17502-048, Gibco) for 72 hours in a 37° C incubator. For ICC, primary antibodies (Nestin (1:500, MA1-110, Thermo-Fisher Scientific, Uppsala, Sweden), Tuj1 (1:1000, ab78078, Abcam, Cambridge, UK), GFAP (1:100, V2129, NSJ Bioreagents, San Diego, CA, USA), and Claudin11 (1:1000, 36–4500, Thermo-Fisher Scientific)) were used.

Polymerase chain reaction (PCR)

Genomic DNA (gDNA) was extracted using a DNeasy® Blood and Tissue Kit (250) (69506, Qiagen, Hilden, Germany) according to the manufacturer’s instructions. Alu PCR was completed using the following primers; forward = 5′-TCAGGAGATCGAG-ACCATCCC-3″ and reverse = 5′-TCCTGCCTCAGCCTCCCAAG-3′ [1] and an Ex Taq DNA-polymerase kit (RR01CM, Takara-Bio, Tokyo, Japan). The reaction mixtures were comprised of 5 µL of 10× Ex Taq Buffer, 4 µL of dNTP mixture, 1 µL (10 pM) of each primer, 2 µL (50ng) of gDNA template, 0.25 µL of TaKaRa Ex Taq, and 36.75 µL of sterile distilled water. Cycling conditions were as follows; 50° C for 20 seconds (s), 95° C for 15 min, 40 cycles of denaturation at 95° C for 15 s, annealing at 62° C for 30 s, and extension at 72° C for 30 s. The PCR product (20 µL) was electrophoresed on a 2% agarose gel with a 100-bp marker (V1002-100, GenDEPOT, Barker, Tx, USA) and the normalized intensity from these gels was quantified using ImageJ software.

SUPPLEMENTARY REFERENCE

1. Szabo S, Jaeger K, Fischer H, Tschachler E, Parson W, Eckhart L. *In situ* labeling of DNA reveals interindividual variation in nuclear DNA breakdown in hair and may be useful to predict success of forensic genotyping of hair. Int J Legal Med. 2012; 126:63–70. https://doi.org/10.1007/s00414-011-0566-5 PMID:21475959
