# Supplementary material for: JAK2/STAT3 pathway mediates neuroprotective and pro-angiogenic treatment effects of adult human neural stem cells in middle cerebral artery occlusion stroke animal models
Source: Aging (Albany NY). 2022 Nov 29;14(22):8944–69. doi: 10.18632/aging.204410 (PMC9740376; doi:10.18632/aging.204410)
Supplement: Supplementary Figures [file aging-14-204410-s002.pdf]

SUPPLEMENTARY FIGURES

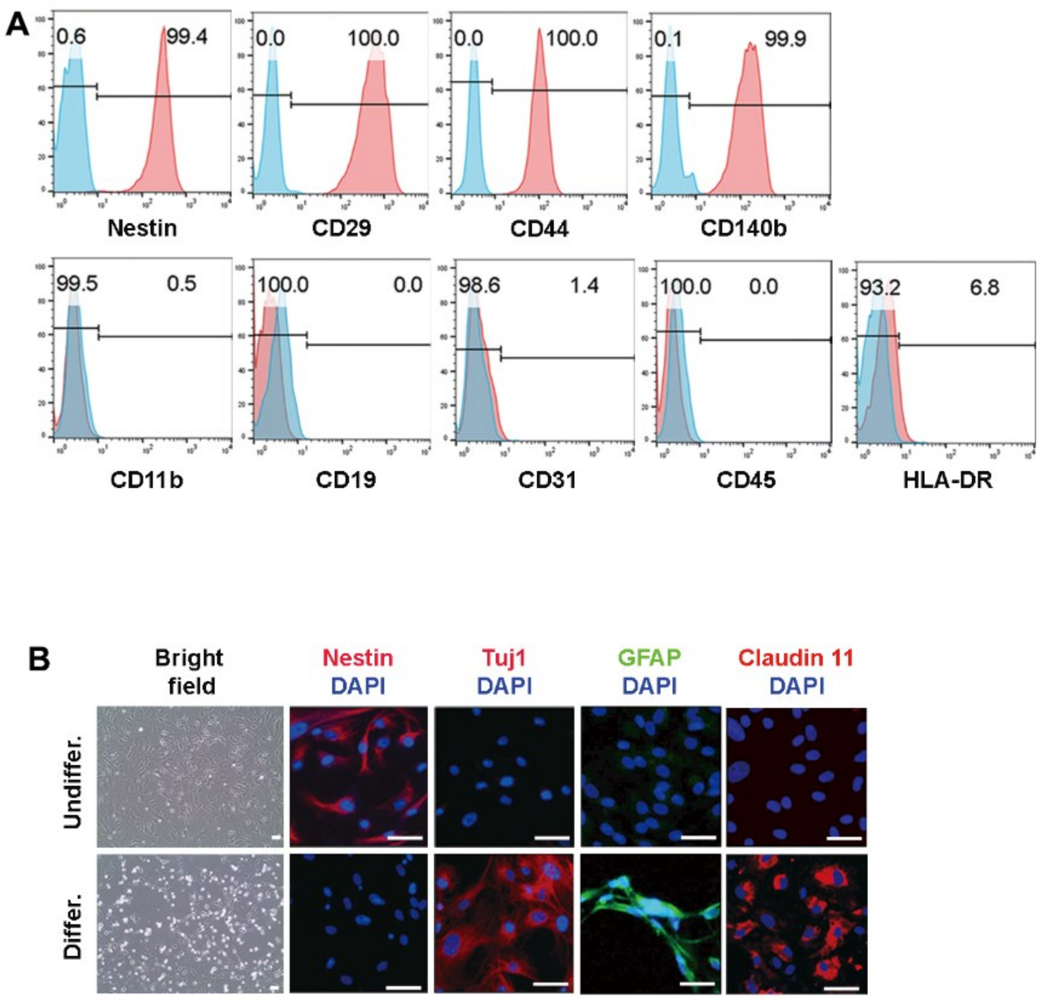

Supplementary Figure 1. Characteristics of ahNSCs. (**A**) Expression of Nestin, CD29, CD44, CD140b, CD11b, CD19, CD31, CD45, and HLA-DR was analyzed by flow cytometry. HLA-DR was used as negative control. Blue = isotype control. Number = percent of stained cells in the area, below. (**B**) The differentiation potential of ahNSCs was tested by ICC. Representative images show bright field, Nestin, Tuj1, GFAP, or Claudin 11 before (Undiffer.) or after differentiation (Differ.). Scale bar = 100 µm.

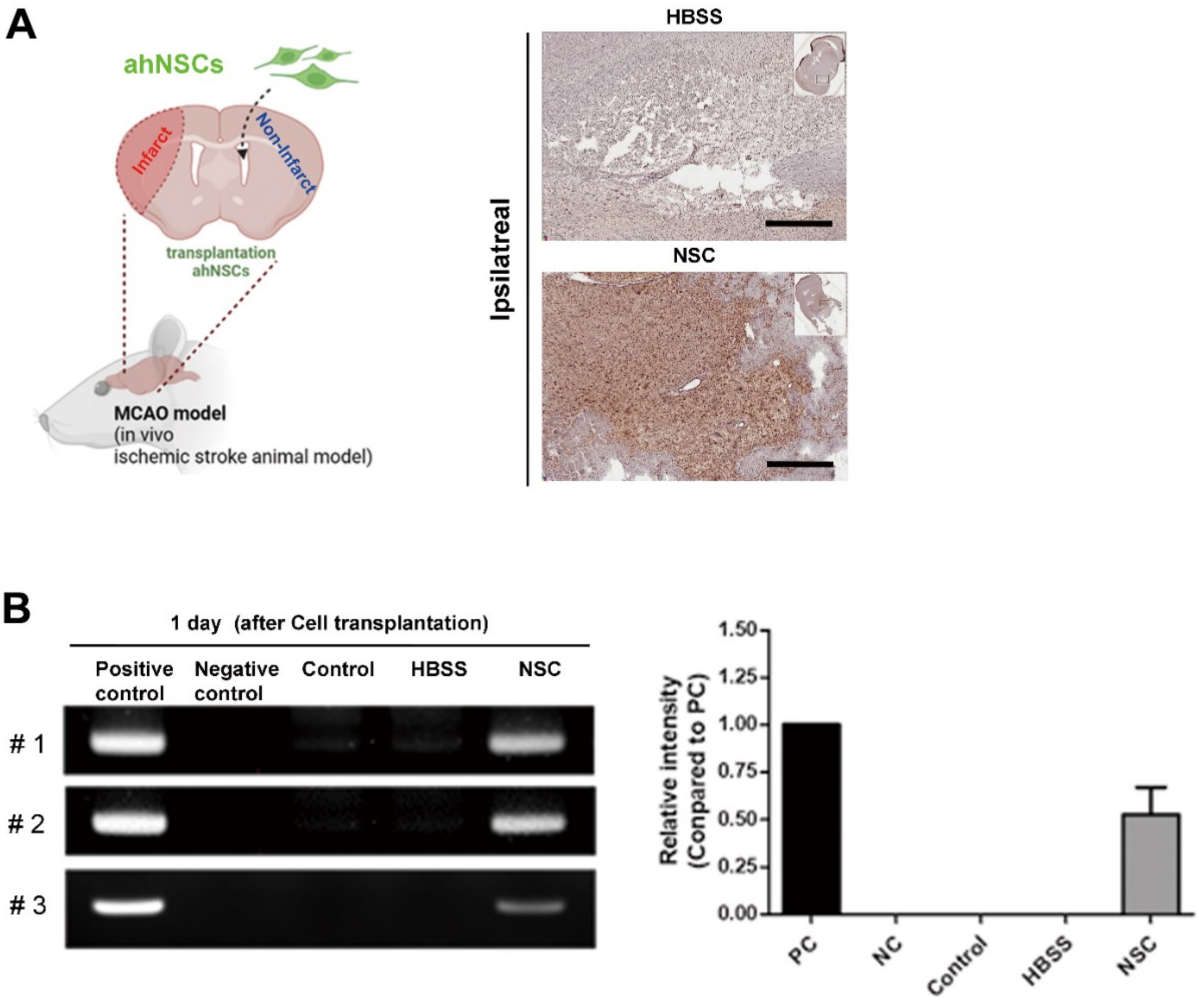

Supplementary Figure 2. Distribution of ahNSCs in the brains of ischemic stroke animal models at 1 day after transplantation. (**A**) AhNSCs were injected into the contralateral (non-infarct) right lateral ventricle (left). Immunohistochemistry against human cytoplasm (right) detected ahNSCs in the ischemic areas of the ipsilateral (infarct) brain hemispheres in the NSC group, while no human cytoplasm-positive cells were found in the HBSS group. n = 3 for each group. Scale bar = 600 µm. (**B**) The presence of ahNSCs was detected by PCR of human specific-Alu sequences in the ipsilateral (infarct) brain hemispheres. Human specific-Alu sequences were amplified only in the NSC group (left). Positive control (PC) = human mesenchymal stem cells, negative control (NC) = distilled water. n = 3 for each group. Relative intensities of the PCR products were quantified using Image J software (right). Data = mean ± SEM.

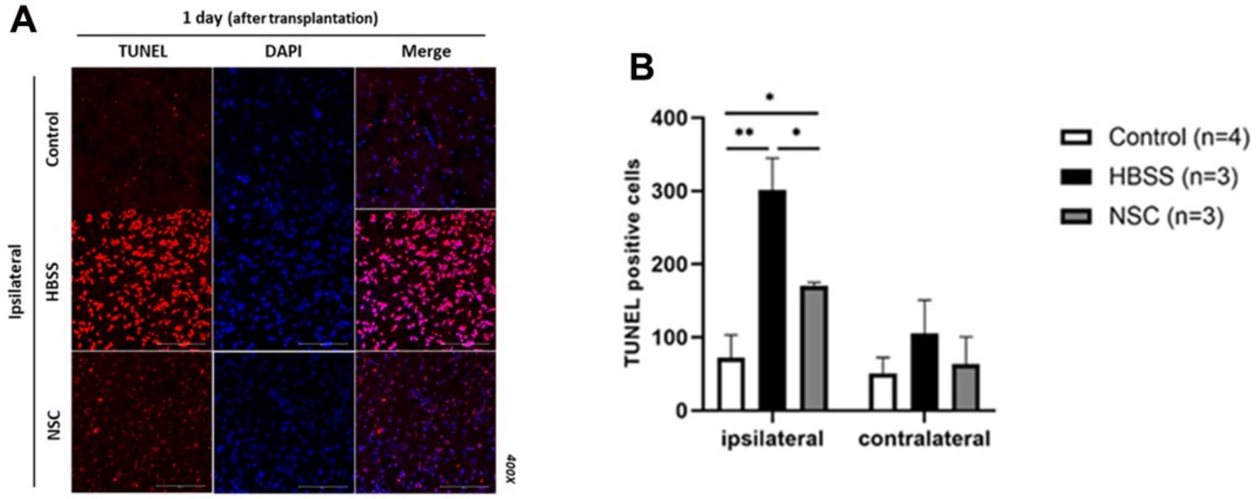

Supplementary Figure 3. *In vivo* neuroprotective activities of ahNSCs. (**A**) TUNEL staining was performed at 1 day after ahNSCs transplantation in the brains of ischemic stroke animal models. Representative images show the results of TUNEL staining (red) in the ipsilateral (infarct) hemisphere of each group (n = 4 for the Control group, n = 3 for the HBSS group, n = 3 for the NSC group). Blue = nuclei. Scale bar = 100 μm. (**B**) Numbers of TUNEL-positive cells were calculated and then compared among the groups. Mean ± SEM. * P < 0.05; ** P < 0.01.

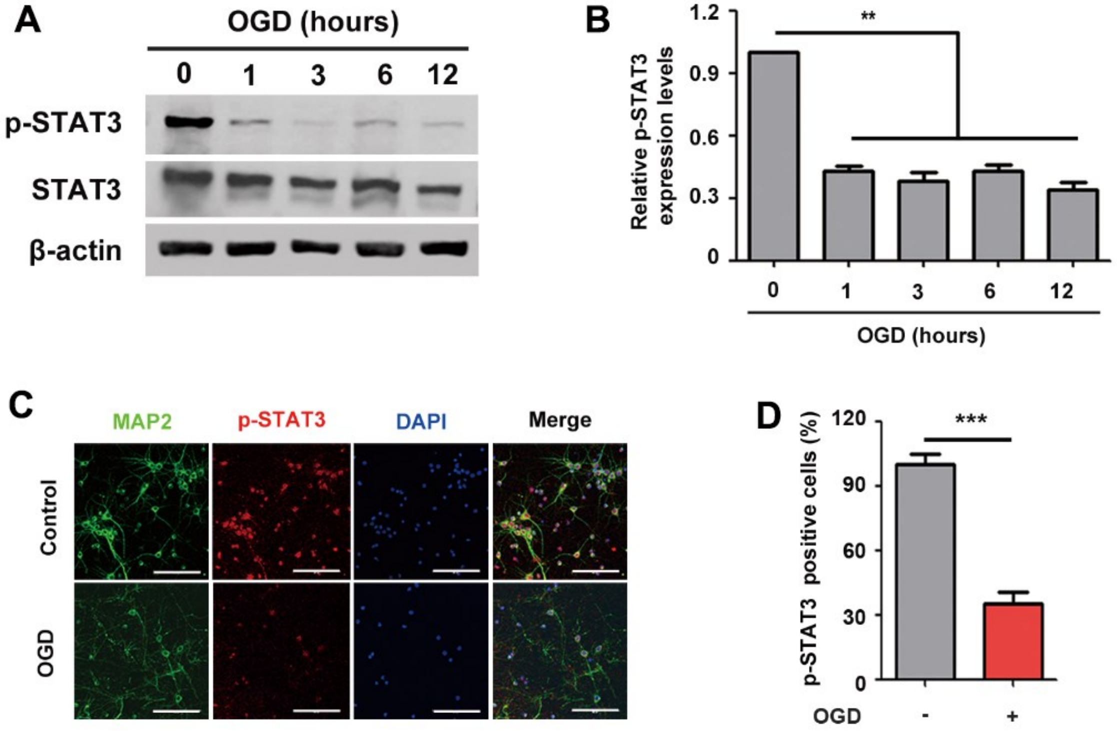

Supplementary Figure 4. Effects of OGD condition on phosphorylation of STAT3 of primary cortical cells. (**A**) Expression of p-STAT3 and STAT3 (79/86 kDa) of primary cortical neurons (n = 3 per group) was accessed after application of OGD condition (0, 1, 3, 6, and 12 h) by western blot analysis. The pictures show representative images. β-actin (43 kDa) = loading control. (**B**) Expression of p-STAT3 was quantified and then compared. Mean ± SD. ** *P* < 0.01. (**C**) Representative images of immunofluorescence staining against MAP2 and p-STAT3 of primary cortical neurons (n = 3 per group). Scale bar = 100 μm. (**D**) Percent of p-STAT3-positive cells was determined and compared. Mean ± SD. *** *P* < 0.001.
