# Supplementary material for: JAK2/STAT3 pathway mediates neuroprotective and pro-angiogenic treatment effects of adult human neural stem cells in middle cerebral artery occlusion stroke animal models
Source: Aging (Albany NY). 2022 Nov 29;14(22):8944–69. doi: 10.18632/aging.204410 (PMC9740376; doi:10.18632/aging.204410)
Supplement: Supplementary Table 1 [file aging-14-204410-s003.pdf]

SUPPLEMENTARY TABLE

Supplementary Table 1. Primer pairs used for qRT-PCR analysis.

Gene	Accession no.	Forward primer	Reverse primer
OSMR	NM_001005384.1	GGTGCTACAAAAGCCCACAT	TGTCTGGCTCCAGATCACTG
Npas4	NM_153626.1	GGTGAGACTTCAAGCCAAGC	AGTGCTCCTGGGAGAAGACA
Stat3	NM_012747.2	TCACTTGGGTGGAAAAGGAC	TGGGAATGTCAGGGTAGAGG
Socs2	NM_058208.2	GACGGGAAATTCAGATTGGA	AATGCTGAGTCGGCAGAAGT
Socs3	NM_053565.1	CCTTTGAGGTTCAGGAGCAG	CGTTGACAGTCTTCCGACAA
Fgf10	NM_012951.1	GAGATGTCCGCTGGAGAAAG	CCCCTTCTTGTTCATGGCTA
Gadd45g	NM_001077640.2	GCTGGCCATAGACGAAGAAG	TGACGCTTGCTGACCAGGAG
Gfap	NM_017009.2	GGCGAAGAAAACCGCATCACCATTCCTGTA	TCGACTCCTTAATGACCTCGCCATCCCGCA
Actb	NM_031144	TTTGAGACCTTCAACACCCC	ATAGCTCTTCTCCAGGGAGG
